# Knowledge, Perspectives, and Risks Perceptions on Gene Drive and Genetically Modified Mosquitoes for Malaria Control among African Stakeholders: A Scoping Review Protocol

**DOI:** 10.21203/rs.3.rs-6048767/v1

**Published:** 2025-07-09

**Authors:** Abou Sogodogo, Samba Diarra, Soumba Keita, Idiatou Diallo, Sarah Hartley, Nouhoum Telly, Housséini Dolo, Daouda Sanogo, Fousseyni Kané, Cheick Oumar Tangara, Mahamadou Diakité, Oumar Sangho, Kassoum Kayentao, Hannah Fritz, Peter J. Winch, Seydou Doumbia

**Affiliations:** University Clinical Research Center/University of Sciences Techniques and Technologies of Bamako; University of Clinical Research Center/University of Sciences Techniques and Technologies of Bamako; University Clinical Research Center/University of Sciences Techniques and Technologies of Bamako; Departement of Global Health and Population:Harvard University T H Chan School of Public Health; Departement of Science, Innovation, Technology and Entrepreneurship:University of Exeter; Departement of Education and Research in Public Health and Specialities:University of Sciences Techniques and Technologies of Bamako; Departement of Education and Research in Public Health and Specialities:University of Sciences Techniques and Technologies of Bamako; Univertisy Clinical Research Center/University of Sciences Techniques and Technologies of Bamako; University Clinical Research Center/University of Sciences Techniques and Technologies of Bamako; University Clinical Research Center/University of Sciences Techniques and Technologies of Bamako; University Clinical Research Center/University of Sciences Techniques and Technologies of Bamako; Departement of Education and Research in Public Health and Specialities/University of Sciences Techniques and Technologies of Bamako; Malaria Research and Training Center/University of Sciences Techniques and Technologies of Bamako; Departement of International Health/ Johns Hopkins Bloomberg School of Public Health; Departement of International Health/Johns Hopkins Bloomberg School of Public Health; University Clinical Research Center/University of Sciences Techniques and Technologies of Bamako

**Keywords:** stakeholders, perspectives, risks perceptions, knowledge, Africa, gene drive mosquitoes, genetically modified mosquitos, malaria

## Abstract

**Objective:**

The objective of this scoping review is to understand the knowledge and perspectives of African stakeholders regarding the use of gene drive mosquitoes in malaria control.

**Introduction:**

The prospect of using gene drive and genetically modified mosquitoes as tools for malaria control is generating considerable debate, particularly with regard to its acceptance and implications among key stakeholders, as well as the nature of governance established in its management. This study aims to fill the gaps in understanding the views of African stakeholders on the risks associated with these mosquitoes.

**Inclusion criteria:**

This review will include scientific articles and grey literature that explore the knowledge, perspectives, and risks perceptions of African stakeholders on the use of gene drive and genetically modified mosquitoes. Exclusions will apply to documents with restricted access, those addressing diseases other than malaria, and those involving stakeholders outside Africa.

**Methods:**

The search will be conducted across PubMed, Embase, Science Direct, Cochrane, and Google Scholar using index terms and keyword strategies tailored to each database. Documents will be selected in four stages: identification, duplicate removal, title and abstract screening, and full-text review for final inclusion. Screening will be conducted on Rayyan by two independent reviewers, with references managed in Zotero. Data extraction will include details on authorship, publication year, study objectives, design, methodology, sample size, findings, conclusions, and limitations. A thematic analysis of the extracted data will be conducted.

**Discussion:**

This scoping review will provide a clear understanding of the knowledge, perspectives, and risks perceived by African stakeholders regarding the use of gene drive and genetically modified mosquitoes in malaria control. The results will help to improve communities’ engagement, which is crucial to the success of this technology.

**Registration:**

This protocol is registered on the Open Science Framework (OSF) (https://osf.io/4kz85).

## Introduction

Malaria is a disease caused by a parasite of the genus *Plasmodium*, transmitted to humans through an infective bite by the female Anopheles mosquito. According to the World Health Organization (WHO), there were an estimated 263 million cases of malaria and 597,000 malaria-related deaths worldwide in 2023 [1]. Africa accounts for 94% of cases and 95% of deaths due to the disease [1]. Current strategies such as insecticide-treated mosquito nets, indoor residual spraying, and antimalarial drugs face the challenge of insecticide and antimalarial drug resistance [2, 3].

Gene drive mosquito and genetically modified mosquito technologies offer the possibility of overcoming current challenges and moving towards eliminating malaria. Genetically modified mosquitoes (GMM) are mosquitoes that have heritable traits introduced by recombinant DNA technology that alter the strain, line, or colony in a manner usually intended to reduce the transmission of mosquito-borne human diseases such as malaria [4]. Hereditary markers can be introduced into GMMs to facilitate monitoring after their release into the environment [5]. GMMs is designed to persist for a few period of time (selt-limiting), they are not expected to persist in the environment or to spread far beyond the release site [5]. Whereas gene drive refers to a phenomenon observed in sexually reproducing organisms, it’s a process based on preferential inheritance where a gene is passed on from parent to offspring at a greater than Mendelian rate [6]. Most or all of the offspring receiving the driving genetic element, enabling the modification to spread rapidly throughout the population, even if they are released from a low initial frequency [7, 8]. The modification is transmitted by interbreeding populations of wild mosquitoes of the target species and persists indefinitely within the local mosquito population [5]. This involves higher ecological concerns due to long-term effects [5, 9]. Studies have demonstrated the efficacy of these technologies in preventing mosquitoes from transmitting the malaria parasite or suppressing the mosquito population [10]. Research suggests that these technologies have considerable potential benefits, particularly for countries where malaria is endemic and limited resources are available to combat the disease [9].

Despite its potential advantages, genetic modification is controversial. The Civil Society Working Group on Genetic Modification unsuccessfully called for a moratorium on genetic guidance research at the 13th and 14th Conferences of the Parties to the Convention on Biological Diversity in Mexico (2016) and Egypt (2018) [11, 12]. Concerns about ecological risks and uncertainties were expressed, given that gene drive mosquitoes are designed to spread through a population and persist in the environment [13]. Using gene drive or GMM often raises questions about the risks to humans, animals, and the environment [14–16]. A procedure for testing the use of gene drive mosquitoes in stages is thus needed [17, 18]. Specifically, there is a strong need for progressive testing, ranging from laboratory trials to ongoing monitoring after release [9].

Over the years, several consortia of researchers around the world have been involved in developing the technology of gene drive and GMM [19, 20]. Africa, bearing the largest burden of malaria, is the main target for gene drive and genetically modified mosquitoes. Several African countries, including Mali, Burkina Faso, Uganda, Ghana, and Cape Verde are involved in developing the technologies [21]. Burkina Faso carried out the first community trial of Genetically modified mosquitoes in Africa [21, 22]. Many calls have been made by scientists and funding agencies for public participation at all stages of the process [23, 24]. The 2016 report from the National Academies of Sciences, Engineering and Medicine identified public engagement as a key area of responsible science [25]. Most governance documents for genetic modification in global health emphasize stakeholder engagement for normative and instrumental justifications [9, 24–26]. The WHO, the African Union (UN), and other organizations insist on the involvement of all stakeholders at all levels [27–29]. Any effective approach to vector-borne disease control requires a strong and meaningful commitment from communities and other stakeholders [4]. Stakeholder engagement is considered “essential to meeting ethical obligations of informed consent, building trust and gaining acceptance for research” [9]. Similarly, studies examining stakeholder options are underway in the African region prior to large-scale implementation [21]. To this end, several studies have been carried out in different African countries to understand stakeholders’ perceptions, knowledge, and perspectives on the use of gene drive and genetically modified mosquitoes [9, 15, 27–32]. From these studies, we can say that there has been very good engagement in African countries over the last six or seven years, but the majority of studies have often focused on a single community in a single country or on a single distinct stakeholder group. This work aims to evaluate and map the findings of studies on gene drive and genetically modified mosquitoes, identifying commonalities and gaps.

A preliminary search of databases, including PubMed, Cochrane, and Science Direct, identified no existing reviews that address stakeholder perspectives or recommendations related explicitly to gene drive and genetically modified mosquitoes in Africa. This gap in literature underscores the need to focus on stakeholder-related issues, which are critical for successful gene drive and genetically modified mosquitoes’ implementation [21]. However, a clear understanding of stakeholder knowledge, perceptions, and concerns is essential to guide gene drive and genetically modified mosquito release programs and foster community engagement. In this context, stakeholders include community members, researchers, policymakers, and public health officials involved in, or affected by, the development and deployment of gene drive and genetically modified mosquitoes.

Given the emerging nature of gene drive and genetically modified mosquito technologies and the scarcity of consolidated evidence on stakeholder perspectives, a scoping review is the most appropriate method. The choice to perform a scoping review instead of a systematic review arises from the necessity to capture the variety and scope of research in this developing field [33]. A scoping review effectively synthesizes diverse studies, methodologies, and contexts, revealing gaps, trends, and unresolved questions critical to the intricate and dynamic landscape of gene drive and genetically modified mosquito research in Africa [34, 35]. In contrast to systematic reviews, which concentrate on specific inquiries, this method facilitates a thorough examination of the literature landscape, laying the groundwork for future focused studies and evidence-based policies [33, 36]..

The objective of this scoping review is to understand the perceptions and perspectives of African stakeholders regarding the use of gene drive and genetically modified mosquitoes in malaria control through a mapping of the existing literature.

## Review questions

This review will be conducted in an effort to answer the following key questions:

- What is the extent of the literature on the risks of implementing gene drive and genetically modified mosquitoes in combating malaria as perceived by African stakeholders?
- What are the perspectives of African stakeholders regarding the introduction of gene drive and genetically modified mosquitoes in malaria control?
- What are the recommendations of African policymakers regarding the introduction of gene drive and genetically modified mosquitoes for malaria control?
- What are the gaps in the literature on the introduction of gene drive and genetically modified mosquitoes in the fight against malaria in Africa?

## Inclusion criteria

### Participants

This review will include studies that focus on stakeholders involved in, or affected by, the use of gene drive and GMMs in malaria control within Africa. Stakeholders include local communities, researchers or academics, and policymakers. Studies involving participants from other continents or unrelated groups will be excluded.

### Concept

The review will include studies and documents addressing knowledge, perceptions, attitudes, or risk assessments related to the use of gene drive mosquitoes or GMM in malaria control. Studies focusing on genetic modification for diseases other than malaria or lacking relevant concepts will be excluded.

### Context

The context for this scoping review is Africa. Studies conducted within African countries where malaria is a public health concern will be included. However, studies from countries in Africa that have officially eliminated malaria (e.g., Algeria, Lesotho, Mauritius, Seychelles, Egypt, Libya, Morocco, and Tunisia) will be excluded to ensure the review focuses on contexts where gene drive mosquitoes or GMM are actively relevant to malaria control efforts.

### Types of sources

This scoping review will consider a broad range of study designs and sources, including observational study designs such as cross-sectional studies, case reports, case series, case-control studies, and cohort studies. Experimental and quasi-experimental studies, including randomized controlled trials, non-randomized controlled trials, and pre-post studies, will also be included. Additionally, the review will include descriptive observational studies, qualitative studies such as those employing phenomenology, grounded theory, ethnography, or qualitative description, and research methods like action research and feminist research. Texts and opinion papers, conference abstracts, theses, dissertations, and decisions or policy documents related to gene drive mosquitoes or GMMs in malaria control will also be considered as part of the review.

## Methods

The proposed scoping review will be conducted following the JBI methodology for scoping reviews [37,38] and the Preferred Reporting Items for Systematic Reviews and Meta-Analyses extension for Reviews (PRISMA-ScR) [39]. The PRISMA-ScR contains a 20-item checklist that is aimed at facilitating the development and reporting of robust scoping review protocols [39]. Any changes to this protocol will be tracked and reported in the final review to promote transparency and reproducibility. The current protocol was drafted following the JBI methodology for protocols of scoping reviews and an adapted PRISMA-ScR checklist (**Appendix #1**). The protocol for this review was also registered in the Open Science Framework ((https://osf.io/4kz85).

### Search strategy

The search strategy aims to locate both published and unpublished studies. A three-step search strategy will be utilized in this review. We will carry out an initial search on PubMed. Keywords contained in titles and abstracts, as well as MeSH terms describing relevant articles, will be used to develop a comprehensive search strategy. The complete search strategy for PubMed is detailed in (**Appendix #2**). This strategy will be adapted for each included database and information source, such as Google Scholar and additional grey literature sources. The reference lists of all included sources of evidence will be screened for additional studies, including conference abstracts, theses, and dissertations. The following keywords will be used: (“perspective” OR “knowledge” OR “perceptions” OR “recommendation”) AND (“gene drive mosquitoes” OR “genetically modified mosquitoes”) AND “malaria” AND “African countries”. The search will use Boolean operators “AND” and “OR,” with keywords and indexing terms expanded through synonym searches and web-based sources. The search strategy will not be restricted by language or year to ensure comprehensive coverage. The snowballing technique and additional web-based searches will supplement database searches, and the search strategy will be reviewed before the final implementation.

### Study/Source of evidence selection

The selection of studies and documents will be carried out in four stages: (1) identification of studies and downloading of citations from electronic databases and grey literature sources, including PubMed, Embase, ScienceDirect, Cochrane Library, and Google Scholar; (2) removal of duplicates using Zotero software; (3) screening of titles and abstracts against inclusion criteria by two independent reviewers in Rayyan [40]; and (4) screening of full texts in detail against inclusion criteria by two independent reviewers.

Throughout the process, any disagreements will be resolved through discussion or by involving a third reviewer. Reasons for excluding full-text sources of evidence will be recorded and reported in the final scoping review. The PRISMA flow diagram will be used to present the search results and study inclusion process (available at: https://www.prisma-statement.org/prisma-2020-flow-diagram).

### Data extraction

Data will be extracted from selected sources of evidence by two independent reviewers using a pretested data extraction tool (**Appendix #3**). This tool includes fields for key study details such as the first author, year of publication, study title, objectives, methodology, sample size, type of study, main findings, conclusions, and limitations. Grey literature sources will also be assessed using this tool, with modifications to accommodate their unique formats.

The data extraction tool will be piloted before formal use to ensure its suitability. Modifications will be made as needed during the extraction process, and any changes will be documented in the final report. Disagreements in data extraction will be resolved through discussion or by involving a third reviewer. When necessary, the authors of included studies will be contacted to request missing or additional data.

### Data analysis and presentation

Due to the potential heterogeneity of included studies in terms of design, results, and quality, a thematic analysis will be performed. Data will be grouped by study type to facilitate a structured synthesis. Quantitative data will be summarized in tables and diagrams created using Excel 2013, while qualitative data will be synthesized narratively. The results will include a descriptive overview of the evidence and address the review objectives and questions. The narrative summary will accompany the tabular and graphical presentations, explaining how the findings relate to the knowledge and perceptions of GMM-related risks in malaria control in Africa.

### Expected results

This scoping review will provide a clear understanding of the knowledge, perspectives, and risks perceived by African stakeholders regarding the use of gene drive and genetically modified mosquitoes in malaria control. It will provide the recommendations of African policymakers regarding the introduction of gene drive and genetically modified mosquitoes for malaria control. The results will help to improve communities’ engagement, which is crucial to the success of this technology.

## Supplementary Files

This is a list of supplementary files associated with this preprint. Click to download.

- Appendixes.docx
